# Supplementary material for: DMRforPairs: identifying Differentially Methylated Regions between unique samples using array based methylation profiles
Source: BMC Bioinformatics. 2014 May 15;15:141. doi: 10.1186/1471-2105-15-141 (PMC4046028; doi:10.1186/1471-2105-15-141)
Supplement: Additional file 2 — DMRforPairs output for the comparison of A431-MCF7 and NA17018-NA17105. Please start from the HTML files in each folder. Available via the BMC Bioinformatics website. [file 1471-2105-15-141-S2.zip › 1394847754114233_MOESM2_ESM/A431_MCF7/figures/3947.pdf]

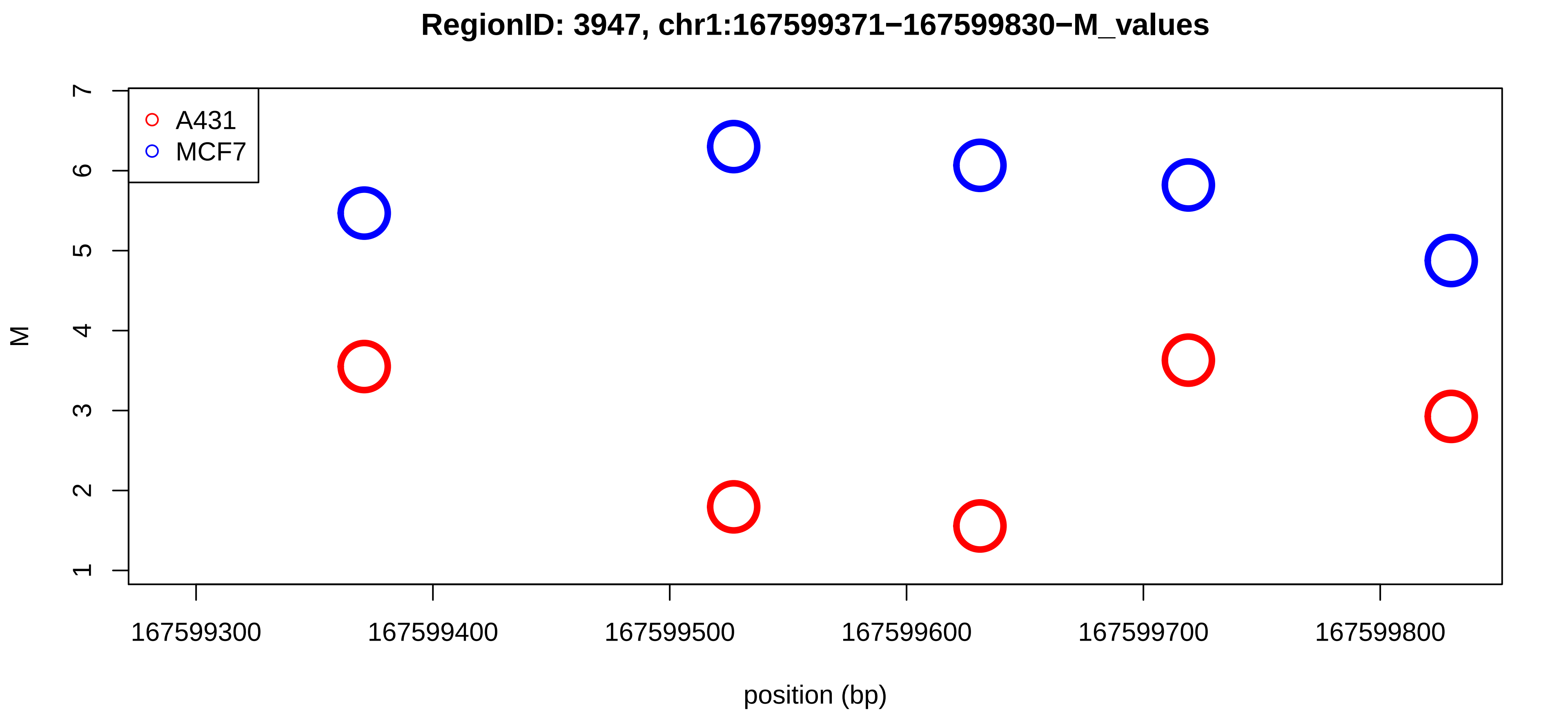
RegionID: 3947, chr1:167599371−167599830−M_values
A431
MCF7
M
1
2
3
4
5
6
7
167599300
167599400
167599500
167599600
167599700
167599800
position (bp)

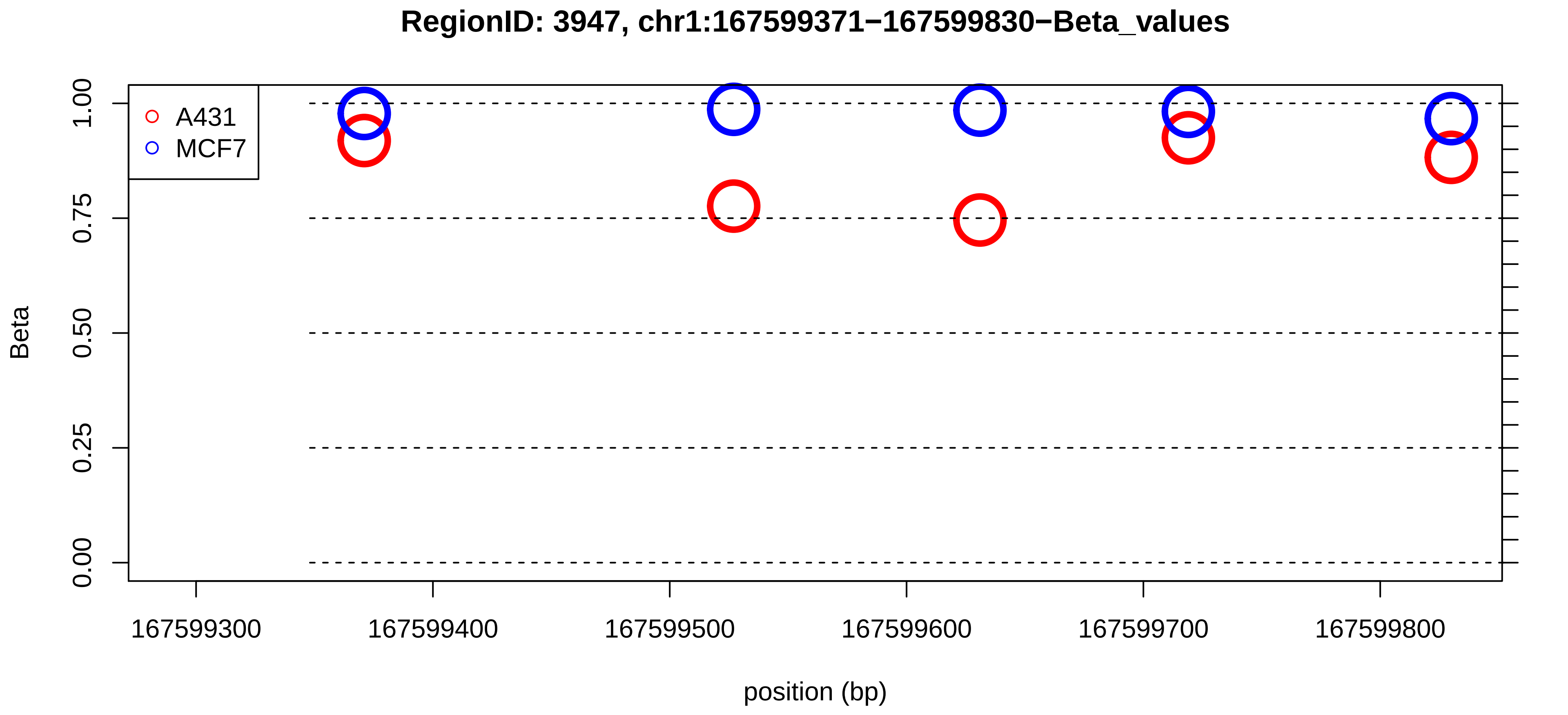
RegionID: 3947, chr1:167599371−167599830−Beta_values
A431
MCF7
Beta
0.00
0.25
0.50
0.75
1.00
167599300
167599400
167599500
167599600
167599700
167599800
position (bp)

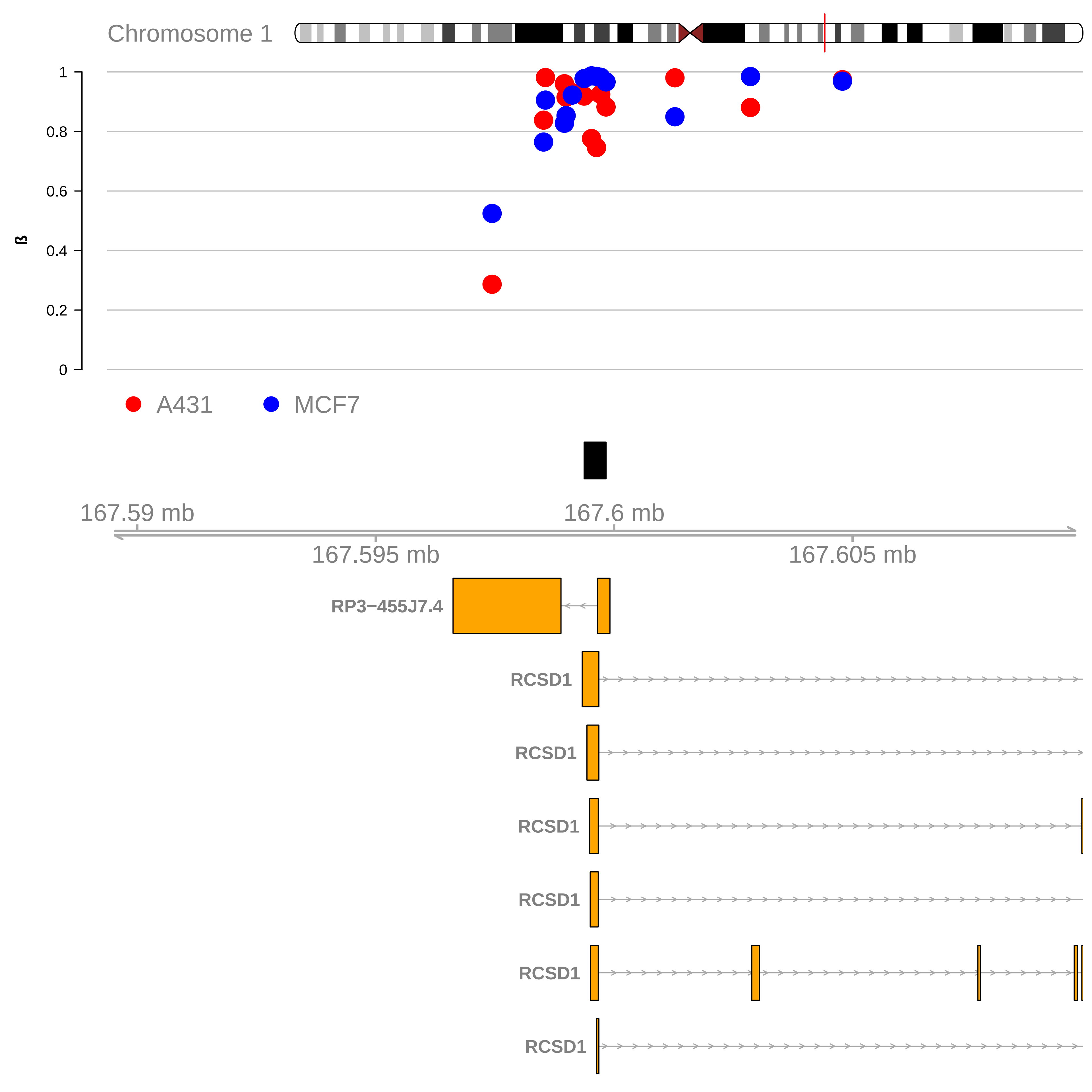

Chromosome 1
1
0.8
0.6
0.4
0.2
0
β
A431
MCF7
167.59 mb
167.6 mb
167.595 mb
167.605 mb
RP3−455J7.4
RCSD1
RCSD1
RCSD1
RCSD1
RCSD1
RCSD1
